# High Mobility Group Box 1 Protein Level as a Novel Biomarker for the Development of Peri-Implant Disease

**DOI:** 10.1038/s41598-017-06937-y

**Published:** 2017-08-01

**Authors:** Jinpan Liu, Ronghui Li, Tongjun Liu, Xiaohui Rausch-Fan, Mingguo Wang

**Affiliations:** 1 Department of Stomatology, Jinan Central Hospital, Shandong University, Jinan, 250013 P.R. China; 2 Division of Conservative Dentistry and Periodontology, School of Dentistry, Medical University of Vienna, Sensengasse 2a, 1090 Vienna, Austria

## Abstract

Peri-implant disease is a chronic inflammation of the soft and hard tissues around a dental implant, resulting from bacterial infection. Recent evidence indicates that some pro-inflammatory cytokines and chemokines released by immunocytes are substantially responsible for the progress and consequence of inflammation. High mobility group box 1 (HMGB1) is released into the extracellular matrix and acts as a key pro-inflammatory factor during injury, necrosis and inflammation. A higher concentration of HMGB1 has been found in gingival crevicular fluid from inflammatory gingival tissue than from healthy sites. HMGB1 mRNA and protein are overexpressed in murine periodontal ligament fibroblasts stimulated with lipopolysaccharide (LPS) and IL-1β. Thus, this study sought to assess HMGB1 expression in peri-implant crevicular fluid (PICF) at each stage of peri-implant disease and to investigate the correlation between HMGB1 and peri-implant disease progress. The results demonstrated that the HMGB1 expression level in PICF is indicative of the progress of peri-implant disease and hence may be a useful diagnostic and prognostic biomarker for peri-implant tissue.

## Introduction

Peri-implant disease is a chronic inflammation of soft and hard tissue around a dental implant, resulting from a bacterial infection. This disease is classified into peri-implant mucositis (MU) and peri-implantitis (PI)^1^, depending on the severity of the lesion. MU is a reversible inflammation restricted to the mucosa, whereas PI is an irreversible inflammation associated with the loss of the supporting marginal bone^1, 2^. If MU is untreated or is not treated in a timely manner, it may develop into PI, and severe effects on the implant may result if PI induces rapid bone loss and causes implant failure.

Contemporary peri-implant pathological models use immunobiology concepts to explain the disease development process, which is similar to that of gingivitis and periodontitis^3, 4^. Plaque is the agent initiating inflammation. Bacterial products, such as lipopolysaccharide (LPS) and endotoxin, recruit neutrophils to the peri-implant pocket, which act as phagocytes and remove bacteria. However, if there is an overload of microbial plaque, the toxic enzymes released from the neutrophils will damage.

Recent evidence has indicated that some pro-inflammatory cytokines and chemokines released by immunocytes are substantially responsible for the progress and consequences of inflammation. High mobility group box 1 (HMGB1) is a nonhistone DNA-binding nuclear protein that plays a crucial role in DNA binding, transcription and repair. HMGB1 is released into the extracellular matrix and act as a key pro-inflammatory factor during injury, necrosis and inflammation^5, 6^. Extracellular HMGB1 mediates the inflammatory response by binding to receptor for advanced glycation end-products (RAGE), toll-like receptor (TLR)-2, TLR-4 and TLR-9, which have been implicated in inflammatory processes^7, 8^. HMGB1 plays an important role in many acute and chronic inflammatory conditions, such as rheumatoid arthritis, osteoarthritis, cancer, lupus and periodontitis^9–13^.

Only a few studies have investigated the role of HMGB1 in the pathological process of periodontitis. A higher concentration of HMGB1 has been found in gingival crevicular fluid from inflammatory gingival tissue compared with healthy sites^14^. Additionally, HMGB1 mRNA and protein are overexpressed in murine periodontal ligament fibroblasts stimulated with LPS and interleukin (IL)-1β^12^. These observations suggest that HMGB1 plays a role in periodontitis. However, the mechanism by which HMGB1 participates in periodontitis and peri-implant disease is still unclear.

Several pro-inflammatory factors have been found to be related to the progress of periodontitis^15, 16^, such as IL-1β, IL-6, IL-8 and tumor necrosis factor (TNF)-α. In experimental models, the higher expression of HMGB1 combined with IL-1β and TNF-α results in alveolar bone loss in periodontitis^12^.

Although there have been numerous studies on the role of inflammation in periodontitis, fewer studies have investigated the role of HMGB1 in the progress of peri-implant disease, and no studies have been conducted on the association between HMGB1 and clinical parameters, such as plaque index, bleeding index, pocket probing depth, and gingival index. Furthermore, the correlation between HMGB1 and other pro-inflammatory cytokines also remains to be evaluated.

Thus, the aim of the present study was to investigate the association between the expression of HMGB1 and the progress of peri-implant disease and clinical parameters. In addition, the study also evaluated the correlation between HMGB1 and levels of IL-1β, IL-6, IL-8, and TNF-α.

## Materials and Methods

### Study population and specimen collection

The study population included 79 implant sites from 47 healthy adult volunteers (26 females and 21 males), 18–59 years of age (mean age 43 ± 11.7 years). Patients were treated with endosseous titanium implants with a platform-switching design (DIO-IFN, DongSeo Inc., Korea). From those selected 79 dental implants, 20 dental implants were placed at maxillary premolar and 15 were placed at maxillary molar regions, whereas 25 dental implants were placed at mandibular premolar and 19 were placed at mandibular molar regions.The implants were 3.8–5.0 mm in diameter and 8.5–13.0 mm in length and were inserted into type II or type III bone. The mean time in function was 65.2 ± 4.7 months (range, 57 to 71 months).

A thorough medical and dental history was taken for each patient. The criteria for inclusion were as follows: (i) the absence of allergies or metabolic bone disorder; (ii) do not have autoimmune diseases and systematic disease; (iii) nonsmoking status and not drug or alcohol abuse; (iv) not-pregnant; (v) had not used antibiotics within the previous 6 months; (vi) not having undergone any type of peri-implant therapy in the last 6 months. Exclusion criteria included inadequate oral hygiene, periodontal disease.

All procedures were approved by the Ethics Committee of the College of Stomatology, Shandong University (SDU, Jinan, China), registered by number LW201400101. All methods were carried out in accordance with the Declaration of Helsinki. Prior to the study, all volunteers were informed and then signed a consent form authorizing the examinations.

### Clinical examination and grouping

Clinical examinations were performed by two trained examiners, and results were calibrated among the two examiners. The clinical examinations included an assessment of the modified Sulcus Bleeding Index (mBI) (0, 1, 2, or 3), simplified gingival index (sGI) (0, 1, 2, or 3), probing depth (PD), and modified plaque index (mPI) (0, 1, 2, or 3). Clinical measurements were taken at four sites around the implants (mid-mesial, mid-distal, mid-buccal, and mid-lingual).

The PD was measured by using a pressure-sensitive probe with a probing force of 0.25 N (Hu-Friedy PCPUNC 15 Mfg Co. Inc., Chicago IL). The clinical examination was performed one week before peri-implant crevicular fluid (PICF) collection.

The height of the alveolar bone around the implant was determined by periapical radiograph examination. Radiographs were digitized at 600 dpi using a flatbed scanner and crest bone loss measurements were performed based on these digitized images. Peri-implant bone loss was measured by comparing the bone observed in the radiograph examination with that observed immediately after surgery. Crest bone loss at the mesial and distal aspects of each dental implant was determined by using the implant thread dimensions provided by the implant system manufactures, and bone loss larger than one thread was considered a sign of bone loss.

The implant sites were classified into three groups according to the PD and sGI: a healthy gingival group (HG group), a peri-implant mucositis group (MU group) and a peri-implantitis group (PI group). Dental implant sites with no sign of inflammation (PD < 3, sGI ≤ 1) were placed in the HG group, whereas sites exhibiting signs of inflammation (PD ≥ 3, sGI > 1) without a radiographic sign of bone loss were included in the MU group. Those sites with PD ≥ 3, GI > 1 and a radiographic sign of bone loss composed the PI group.

### Sample collection

PICF samples were obtained from each implant by using sterile standardized paper strips^17^ (PerioPaper, Periocal, Oraflow Inc., Aintyville, NY, USA). Patients were scheduled for PICF collection from 8 am to 10 am to avoid diurnal variations (food, drink, saliva and soft mucinous deposits) that might affect the results. Before collection, the volunteers were restrained for breakfast, and to perform the regular brushing. The samples were collected after delivery of the suprastructure, and all sites had the supragingival plaque were cleaned with sterile cotton rolls. Then the sites were isolated with sterile cotton rolls, and were gently dried in order to reduce contamination with plaque or saliva. Regardless of the PD, paper strips were inserted to a standardized 1-mm depth in a given site for a standard sampling time of 30 seconds without traumatizing the tissues. Strips contaminated with blood or saliva were discarded, and corresponding samples were taken 24 h later in order to reduce deviation, and the results came from the combination of two batches. After collection, paper strips were transported immediately to a Periotron which was calibrated,turned on, and allowed to warm up before PICF sampling. Firstly, a blank paper strip was placed in the device to adjust the reading to zero, then PICF samples were measured with the device, and the units were converted to microliters by a software program.

### Laboratory testing

The concentration of HMGB1, IL-1β, IL-6, IL-8 and TNF-α in PICF was assessed by using commercially available high-sensitivity multiplex map human cytokine immunoassay (R&D, SAD, USA) according to the manufacturer’s instructions. Firstly, a 96-well plate was coated by microsphere beads that carring monoclonal antibodies against target analytes. Secondly, samples and standard fluid were pipetted into the wells and incubated overnight at 4 °C. Then washed the wells with a vacuum manifold, and a mixture of biotinylated secondary antibodies was added. After incubation at 37 °C for 1 h, streptavidin conjugated to the fluorescent protein, R-phycoerythrin was added to the beads and incubated at 37 °C for 30 min. Finally washed the wells in order to remove unbound reagents, sheath fluid was added to the wells and the beads were analyzed in the bead analyzer. Levels of cytokines as low as 0.1 pg/ml could be detected with this technique.

### Statistical analysis

The demographic data were compared with a χ^*2*^ *-*test, and the comparisons of numerical data were performed with a one-way ANOVA and least significant difference (LSD) multiple comparison. The concentrations of HMGB1 in the different stages of peri-implant disease were compared with a one-way ANOVA. Spearman rank correlation coefficients were used to assess the correlation between HMGB1 and the clinical parameters of peri-implant disease, and the correlations between HMGB1 and IL-1β, IL-6, IL-8, and TNF-α in PICF were calculated by Pearson correlation. Differences were considered statistically significant at the level of p < 0.05. All statistical analyses were carried out using the statistical software package SPSS version 13.0.

## Results

### Patient characteristics and clinical results

All of the recalled patients met the inclusion criteria and were included in the study. Among 79 implants, 24 implants showed signs of MU, 16 implants showed signs of PI, and 39 implants were classified in the HG group. The demographic and behavioral data including sex, age, and clinical parameters are summarized in Table 1.

**Table 1:** Comparison of the general demographic characteristics and clinical parameters in different stages of peri-implant disease.

Variable	HG (n = 39) $\bar{{\boldsymbol{X}}}\pm {\boldsymbol{S}}$	MU (n = 24) $\bar{{\boldsymbol{X}}}\pm {\boldsymbol{S}}$	PI (n = 16) $\bar{{\boldsymbol{X}}}\pm {\boldsymbol{S}}$	*P1*	*P2*	*P3*
Age	39.3 ± 9.4	41.9 ± 12.8	45.8 ± 13.2			
Sex (M/F)	6/16	27/15	7/9			
sGI	0.47 ± 0.09	2.11 ± 0.33	2.47 ± 0.51	*	*	
mPI	1.17 ± 0.04	1.76 ± 0.63	2.11 ± 0.41	*	*	
mBI	0.11 ± 0.27	1.58 ± 0.31	2.29 ± 0.43	*	*	*
PD	1.94 ± 0.42	3.66 ± 0.93	5.54 ± 1.13	*	*	*

*P1*: HG vs. MU, *P2*: HG vs. PI, *P3*: MU vs. PI; *p-value ≤ 0.05.

The demographic data were compared with a *χ* ^*2*^ *-*test, and the numerical data were compared with a one-way ANOVA and LSD multiple comparison.

These data showed an increasing tendency of sGI, mPI, mBI and PD accompanied by the progress of inflammation. These parameters were significantly different between the HG and MU groups as well as between the MU and PI groups. Interestingly, there were no significant differences in the sGI or mPI between the MU and PI groups.

### The association between HMGB1 and peri-implant disease progression

Comparison of the HMGB1 concentrations in the PICF from the different groups revealed a significant increase in the HMGB1 concentration as the peri-implant disease progressed (Tables 2 and 3). In addition, Pearson correlation coefficients were calculated to investigate the relationship between HMGB1 and the clinical parameters of peri-implant disease, including sGI, mPI, mBI and PD (Table 4), thus revealing a positive correlation between HMGB1 and these clinical parameters. The strongest correlations were between the HMGB1 concentration and PD and sGI, and a moderate correlation was observed between the HMGB1 concentration and mBI. However, the correlation between the HMGB1 concentration and mPI was not significant. Comparison of HMGB1 concentrations in the different mPI groups showed that the differences between the HG group and MU group and between HG group and PI group were significant, whereas the difference between the MU group and PI group was not significant (Table 5).

**Table 2:** Comparison of the concentration of HMGB1 (pg/ml) in different stages of peri-implant disease.

Group	X	SD	Med	SE	N	F	P
HG	167.3	18.9	167.4	3.1	39	2770.2	<0.05
MU	309.4	45.4	316.6	7.5	24		
PI	962. 2	82.5	987.4	16. 1	16		

*X*, mean; *SD*, standard deviation; *Med*, median; *SE*, standard error; *N*, number; *F, f* value.

The concentrations of HMGB1 in different stages of peri-implant disease were compared with a one-way ANOVA.

**Table 3:** Comparison of the concentration of HMGB1, IL-1β, IL-6, IL-8 and TNF-α in PICF (pg/ml) in different stages of peri-implant disease.

variable	HG (n = 39) $\bar{{\boldsymbol{X}}}\pm {\boldsymbol{S}}$	MU(n = 24) $\bar{{\boldsymbol{X}}}\pm {\boldsymbol{S}}$	PI(n = 16) $\bar{{\boldsymbol{X}}}\pm {\boldsymbol{S}}$	P1	P2	P3
HMGB1	167.3 ± 18.9	309.4 ± 45.4	962.6 ± 82.5	*	*	*
IL-1β	227.7 ± 18.5	483.3 ± 65.9	872.2 ± 114.3	*	*	*
IL-6	144.3 ± 33.1	288.7 ± 45.9	537.2 ± 151.4	*		
IL-8	45.3 ± 5.9	117.4 ± 16.4	178.26 ± 162.5	*	*	
TNF-α	178.3 ± 11.9	289.4 ± 7.4	402.6 ± 24.5	*	*	*

values are presented as the mean ± standard deviation; P *P1*: HG vs. MU, *P2*: HG vs. PI, *P3:* MU vs. PI; *p-value ≤ 0.05.

The means in the different groups were compared using one-way ANOVA and LSD multiple comparison.

**Table 4:** Correlation between the concentration of HMGB1 and the clinical parameters of peri-implant disease.

	N	R	P
PD	79	0.859	<0.05
sGI	79	0.804	<0.05
mBI	79	0.649	<0.05
mPI	79	0.448	<0.05

Spearman rank correlation coefficients were used to assess the correlation between the HMGB1 concentration and the clinical parameters of peri-implant disease.

**Table 5:** Comparison of the concentrations of HMGB1 in different mPI groups.

		X	SD	N	P
HG vs. MU	mPI = 0	177.3	19.4	17	0.01
	mPI = 1	186.5	19.6	23	
	mPI = 2	299.3	30.1	19	
	mPI = 3	330.9	27.8	2	
HG vs. PI	mPI = 0	168.4	17.9	13	0.03
	mPI = 1	455.4	31.7	21	
	mPI = 2	717.2	39.8	22	
	mPI = 3	933.6	117.9	7	
MU vs. PI	mPI = 0	0	0	0	0.222
	mPI = 1	691.7	97.1	6	
	mPI = 2	815.3	108.2	25	
	mPI = 3	951.2	115.6	8	

The differences in the concentration of HMGB1 in the different groups were compared with a one-way ANOVA.

### Association between HMGB1 concentration and IL-1β, IL-6, IL-8, and TNF-α levels

To explore the potential association between HMGB1 and the other pro-inflammatory factors in PICF, the correlation coefficients of the association between HMGB1 concentration and IL-1β, IL-6, IL-8, and TNF-α levels in those three groups were calculated (Table 6). There was a high correlation between the HMGB1 level and the expression of IL-1β and TNF-α and a moderate correlation between the HMGB1 level and the expression of IL-6 and IL-8.

**Table 6:** Correlation coefficients between HMGB1, IL-1β, IL-6, IL-8 and TNF-α in PICF (pg/ml).

	HMGB-1	IL-1	IL-6	IL-8	TNF-α
HMGB-1	1.000	0.757*	0.690*	0.685*	0.793*
IL-1	0.757*	1.000	0.551	0.716*	0.858*
IL-6	0.790*	0.551	1.000	0.784*	0.690*
IL-8	0.685*	0.716*	0.784*	1.000	0.588
TNF-α	0.793*	0.858*	0.690*	0.588	1.000

^*^p-value ≤ 0.05.

The correlations between HMGB1, IL-1β, IL-6, IL-8 and TNF-α in PICF were calculated with Pearson correlation.

## Discussion

Peri-implant disease, similarly to periodontitis, may have a multi-factorial background. Page *et al*. have hypothesized that the progress of periodontitis is dependent on a combination of several factors, including the effects of bacteria and their production, overexpression of pro-inflammatory factors (cytokines and chemokines) in the local microenvironment, lower expression of inflammation inhibitors, and the susceptibility of the host^18^. Thus, the balance of cytokines determines whether inflammation occurs and homeostasis is maintained. Investigators are searching for valid biological diagnostic markers with high sensitivity that can indicate the presence of inflammation before clinical damage occurs.

The biochemical content of PICF, similar to that of gingival crevicular fluid (GCF), is considered to accurately reflect the processes occurring in peri-implant tissue^16, 19^. Therefore, assessing the expression of cytokines in PICF from different disease stages can reveal essential information regarding the disease progress.

HMGB1 is a mediator factor newly identified to be related to the onset and progress of periodontitis. In the present study, we assessed the levels of HMGB1 in PICF of patients with HG, MU and PI. The HMGB1 levels were significantly different between different groups (HG group vs. MU group, HG group vs. PI group, MU group vs. PI group), showing a significant increase as peri-implant disease progressed. Additionally, we assessed the correlation between the HMGB1 level and clinical parameters indicating the severity of the peri-implant disease, such as sGI, mBI, PD, and mPI. There was a close correlation between the HMGB1 concentration and sGI and PD, a moderate correlation between the HMGB1 concentration and mBI, and an insignificant correlation between HMGB1 and mPI, thus suggesting that HMGB1 may play a role in the development of peri-implant disease, especially in the destruction of soft and hard tissue.

Interestingly, the present study demonstrated that there was no significant difference in mPI between the MU group and PI group. This result is consistent with findings from a study by Bhardwaj *et al*., which has compared the clinical parameters between HG, MU, and PI groups^20^. In the present study, the comparison of the concentration of HMGB1 in the different mPI groups revealed significant differences between the HG group and MU group as well as the HG group and PI group but not between the MU group and PI group. Although plaque accumulation is the initial inflammatory factor, these data suggested that the amount of plaque is less correlated with the level of HMGB1 and the tissue destruction, such as bone loss, observed in the late stage of the disease

Several studies have investigated the role of HMGB1 in the dental area; however, most of them have investigated its role in periodontitis rather than in peri-implant disease. In periodontitis, a higher concentration of HMGB1 and more HMGB1-positive cells have been observed in GCF and inflammatory gingival epithelial cells, respectively, than in controls *in vitro* ^14^. Moreover, the expression of HMGB1 is higher in GCF and gingival tissue of chronic periodontitis and generalized aggressive periodontitis patients than in controls^21^. The protein expression of HMGB1 has been shown to be up-regulated after stimulation with LPS or IL-1β in murine periodontal ligament fibroblasts, with LPS and IL-1β in human gingival fibroblasts, and with TNF-α and IL-1β in human gingival epithelial cells^12, 14, 22, 23^. In addition, two studies have demonstrated that the inflammatory signal for periodontitis contributes to the translocation of HMGB1 from the nucleus to the cytoplasm and extracellular matrix in gingival epithelial cells^12, 14^. These data demonstrate that HMGB1 is expressed and released by cells residing in the periodontium and consequently responds to inflammatory signals and participates in the progress of periodontitis.

To our knowledge, no studies have been conducted regarding the role of HMGB1 in the progress of peri-implant disease and the correlation of HMGB1 with clinical parameters of peri-implant disease.

The concentration of IL-1β, IL-6, IL-8 and TNF-α in GCF has been shown to be markedly elevated in periodontitis^1, 16^. Our results demonstrated that higher expression of HMGB1 was accompanied by a higher concentration of IL-1β, IL-6, IL-8 and TNF-α. The close correlation between the levels of HMGB1 and these cytokines suggested that these cytokines may cooperatively promote inflammation in peri-implant disease.

Previous studies have revealed that HMGB1 release from monocytes, macrophages, gingival fibroblasts, epithelial cells, and endothelial cells is stimulated by IL-1β and TNF-α. Consequently, HMGB1 activates the release of IL-1β and TNF-α from monocytes and macrophages^11, 14, 22^. Anti-IL-1β antibodies or the IL-1 receptor antagonist block the pro-inflammatory action of HMGB1 in cultured cells^24^. Additionally, administration of anti-HMGB1 antibody has been found to protect mice against the lethality induced by LPS and to decrease the release of IL-1β and TNF-α^25–27^. An increased amount of HMGB1 has also been observed in the salivary glands of patients with Sjogren’s syndrome, and HMGB1 may act together with IL-1β and TNF-α in forming a feed-forward loop promoting inflammation^28^. In agreement with findings from previous studies, our results revealed a strong correlation between the level of HMGB1 and IL-1β and TNF-α.

IL-6 is a pro-inflammatory cytokine and a central mediator of the acute-phase response. This pleiotropic cytokine stimulates B cell differentiation and T cell activation as well as the production of acute-phase proteins, including c-reactive protein^29^. HMGB1 stimulates the release of IL-6 and IL-11 from human periodontal ligament cells^30^ and also induces the expression of IL-6 and TNF-α in the brain^31^. In addition, HMGB1 is released by macrophages and cardiomyocytes, and this release is followed by an increase in TNF-α and IL-6 expression^31^. In an experimental periodontitis model, the expression profile of HMGB1 has been shown to be similar to those of TNF-α and IL-6, with higher expression of those cytokines occurring at day 7 and 15 followed by a decrease on day 30^12^.

IL-8 is a neutrophil chemotactic factor and an early mediator of periodontitis. Its increased concentration in the PICF of some patients with clinically healthy implants may indicate the onset of inflammation^16, 29^. HMGB1 activates human umbilic vein endothelial cells, which then produce multiple inflammatory cytokines, such as IL-6 and IL-8^32^. An intraperitoneal injection of HMGB1, which induces IL-1β and IL-8, provokes the accumulation of neutrophils in the lung^21^.

Our results were consistent with these previous findings and indicated a close correlation between the cytokines IL-1β, IL-6, IL-8, TNF-α and the progress of peri-implant disease with HMGB1. The data suggested that the cytokines are connected in a network and expand the pro-inflammatory properties of themselves and other cytokines. Although HMGB1 itself has only minimal pro-inflammatory characteristics, it can acquire much greater activity through amplifying the response of other cytokines.

In conclusion, the results obtained in this study indicated that HMGB1 in PICF is a key mediator of several pro-inflammatory cytokines and plays a crucial role in the advance of peri-implant disease. HMGB1 can be considered a new biomarker for the peri-implant condition. Future studies should investigate the mechanism of the interaction of pro-inflammatory and anti-inflammatory cytokines in peri-implant disease. Understanding the intricacies of peri-implant disease should enable novel treatment paradigms and perhaps the development of prevention strategies.
